# *Cistanche tubulosa* Protects Dopaminergic Neurons through Regulation of Apoptosis and Glial Cell-Derived Neurotrophic Factor: *in vivo* and *in vitro*

**DOI:** 10.3389/fnagi.2016.00295

**Published:** 2016-12-16

**Authors:** Qian Xu, Wen Fan, Shui-Fen Ye, Yi-Bo Cong, Wei Qin, Shi-Ya Chen, Jing Cai

**Affiliations:** ^1^Institute of Integrated Chinese and Western Medicine, Fujian University of Traditional Chinese MedicineFuzhou, China; ^2^Department of Internal Medicine, Xiamen Hai Cang HospitalXiamen, China; ^3^Department of Geratology, Longyan First HospitalLongyan, China

**Keywords:** *Cistanche tubulosa*, Parkinson, dopaminergic neurons, apoptosis, GDNF

## Abstract

Parkinson’s disease (PD) is a neurodegenerative disease with the pathological hallmark of reduced nigrostriatal dopamine. In traditional Chinese medicine (TCM) clinical practice, the nanopowder of *Cistanche tubulosa* has therapeutic effects on PD. To identify the therapeutic mechanism, this study tested the protective effect of different doses of MPP^+^-induced toxicity in MES23.5 cells using the MTT assay and in the 1-methyl-4-phenyl-1,2,3,6-tetrahydropyridine (MPTP)-induced PD mice (vehicles). Immunohistochemistry was used to assess cytomorphology and tyrosine hydroxylase (TH) expression. Behavioral tests in vehicles, high performance liquid chromatography (HPLC) tests in dopamine, immunohistochemistry and western blot analysis were used to detect the expression of TH, glial cell line-derived neurotrophic factor (GDNF) and its receptors. Our results demonstrated that the *C. tubulosa* nanopowder improved the viability of MPP^+^-treated cells, increased TH expression and reduced the number of apoptotic cells. It also increased Bcl2 protein expression and suppressed Bax protein expression in MPP^+^-treated cells in a dose-dependent manner. In addition, *C. tubulosa* nanopowder improved the behavioral deficits in vehicle mice, reduced the stationary duration of swimming, enhanced the ability for spontaneous activity and increased the expression of GDNF, the GDNF family receptor alpha (GFRα1) and Ret in cells of the substantia nigra (SN). Furthermore, the protein expression of GDNF, GFRα1 and Ret increased after treatment with different doses of *C. tubulosa* nanopowder, with a significant difference between the high-dose and vehicle groups. The protein expression of Bcl2 and Bax were similar in the *in vivo* and *in vitro*, which suggested that *C. tubulosa* nanopowder has anti-apoptotic effects in neurons.

## Introduction

Parkinson’s disease (PD) is a common neurodegenerative disease occuring in elderly people with the pathological manifestations of loss of dopaminergic neurons in the substantia nigra (SN) due to degeneration. The severity of the disease has been shown to be correlated with dopamine (DA) neuronal cell loss in the SN, which is consistent with the view that the neurodegenerative process progresses over many years before any symptoms appear (Sawle and Myers, 1993). The progressive nature of the disease suggests interesting possibilities for therapeutic intervention by blocking the underlying neurodegenerative process. The search for therapy-induced potent and specific actions of neurotrophic factors on DA neuron survival is therefore of considerable interest.

Neurotrophic factors are essential proteins, including nerve growth factor (NGF), brain derived neurotrophic factor (BDNF) and glial cell-derived neurotrophic factor (GDNF), which promote nerve growth, neurological development, axonal guidance and neuronal function. Among all of the neurotrophic factors that protect and promote the repair of dopaminergic neurons, GDNF has the strongest effects (Hong et al., 2008; Rangasamy et al., 2010; Allen et al., 2013). GDNF has been shown to possess potent neurotrophic effects on DA neurons *in vitro* (Lin et al., 1993) and to exert neuroprotective effects *in vivo*. GDNF has been shown to rescue nigral DA neurons from lesion-induced cell death after surgical- or toxin-induced axotomy in rats (Beck et al., 1995; Kearns and Gash, 1995; Sauer et al., 1995) and partially also after systemic administration of N-methyl-4-phenyl-1,2,3,6-tetrahydropyridine (MPTP) in mice (Tomac et al., 1995). The increased incidence of neuronal apoptosis and reduced protective effects of neurotrophic factors, potentially triggered by various pathological factors, underlie the degeneration of dopaminergic neurons (Holden et al., 2006).

*C. tubulosa* is an herbal medicine originating from several plants of the genus *Cistanche*. It is a major therapeutic option for kidney deficiency syndrome which is closely related to androgen hormones in traditional Chinese medicine (TCM). To date, lots of clinical and basic research of *C. tubulosa* have showed the activities on neurodegenerative diseases. The identification of TCM kidney-tonifying prescriptions in PD treatment may thus provide an alternative clinical treatment for PD. Echinacoside (ECH) is a major bioactive component found in the medicinal herb *C. tubulosa*. Studies have shown the therapeutic effects of glycosides of *Cistanche* and ECH, verbascoside (VER) and icariin (ICA) on Alzheimer’s disease (AD), PD and other vascular dementia patients (Urano and Tohda, 2010; Wang et al., 2013; Wu et al., 2014). Wu et al. (2014) suggested that *C. tubulosa* extracts that contained enough ECH and acteoside ameliorated the cognitive dysfunction caused by Aβ-42 via blocking amyloid deposition, and reversing cholinergic and hippocampal dopaminergic neuronal function. Tao et al. (2015) found that phenylethanoid glycosides from *C. tubulosa* (Ph Gs-Ct) prevented high altitude cerebral edema by decreasing the protein and mRNA expression of AQP4 in the brain tissue of rat models.

Previous studies have shown that Chinese herbal compounds, including the three ingredients of *C. tubulosa*, epimedium and rhizoma polygonati, alleviated damage to dopaminergic neurons and increased levels of dopamine by regulating the expression of neurotrophic factors (Wu et al., 2013). Thus, it is not yet known whether the *C. tubulosa*-induced neuroprotective effects are long-lasting and to what extent rescue of nigral DA neurons by administration of GDNF can afford significant preservation of motoric behaviors of relevance for the symptomatology of PD animal. This study used a TCM kidney-tonifying recipe, *C. tubulosa* nanopowder, that has received a national patent (patent number: 2011103028541) in China and has previously shown a certain therapeutic effect in PD. The present study, therefore, was designed to examine the neuro-protective and regenerative effects of *C. tubulosa* treatment and to investigate the apoptosis on the MES23.5 cells and behavioral deficitive rats, and the regulation of GDNF, as measured by a battery of target tests.

## Materials and Methods

### Materials, Reagents and Equipment

*C. tubulosa* was purchased from Beijing Tong Ren Tang Group, Co., Ltd., Beijing, China. ECH, VER and ICA came from the National Institutes for Food and Drug Control, China. The MES23.5 dopaminergic neuronal cell line was a gift from Professor Biao Chen, Laboratory of Neurobiology, Capital Medical University, Beijing, China. Fifty C57BL/6 male mice (weighing 20–25 g each) were purchased from Shanghai SLAC Laboratory Animal Co., Ltd. (license number: SCXK2012-0002), Shanghai, China.

MPP^+^, MTT and glutamine were purchased from Sigma-Aldrich (Carlsbad, CA, USA); DMEM/F12 medium and fetal bovine serum were purchased from Gibco Co. (Life Technologies, Carlsbad, CA, USA); and MPTP, DA standard and homovanillic acid (HVA) standard were purchased from Sigma-Aldrich (Carlsbad, CA, USA). β-actin, Bax, Bcl2, GDNF, GDNF family receptor alpha (GFRα1) and Ret antibodies were purchased from Cell Signaling Technology, Inc. (Beverly, MA, USA); 3,3’-diaminobenzidine (DAB) staining reagent kit was purchased from Fuzhou Maixin Biotech., Ltd. (Fujian, China); and SDS-PAGE gel sample preparation kit, ultrasensitive enhanced chemiluminescence (ECL) detection kit and bicinchoninic acid (BCA) assay were purchased from Beyotime Institute of Biotechnology (Beijing, China).

This study used the following instruments: ELX800 microplate reader (Bio Tek Winooski, VT, USA); CO_2_ incubator (Heraeus, Hanau, Germany); Gel DOC 2000 gel imaging analysis system, electrophoresis cell and electrophoresis tank (Bio-Rad, Hercules, CA, USA); DU-650 protein analyzer (Beckman Coulter, Inc., Fullerton, CA, USA); 5417R high-speed refrigerated centrifuge (Eppendorf, Hamburg, Germany); SXQM dual planetary ball mill (Changsha, Tencan Powder Technology Co., Ltd, Hunan, China); MM400 mixer mill (Retsch GmbH, Haan, Germany); Agilent 1200 high-performance liquid chromatography (HPLC; Agilent Technologies, Santa Clara, CA, USA); PowerPac Basic electrophoresis, PowerPac Basic transmembrane transfer system, Universal Hood II chemiluminescence imager, S1000 Thermal Cycler RNA reverse transcription system (Bio-Rad); Motic Med 6.0 tissue and cell image analysis system (Motic China Group, Co., Ltd, Xiamen, China).

### Preparation of *C. tubulosa* Nanopowder

*C. tubulosa* was weighed, then purified and dehydrated. After conventional pulverization, *C. tubulosa* fine powder was passed through a 200-mesh sieve and freeze-dried. A temperature-controlled vacuum and high-energy ball mill was used to prepare the *C. tubulosa* nanopowder. First, raw *C. tubulosa* nanopowder was placed in a vacuum ball-milling tank that was loaded with carbide grinding balls. The ratio between the grinding balls and *C. tubulosa* nanopowder ranged from 15:1 to 5:1. To obtain a fine powder, the speed and duration of the high-energy ball mill were set to 300 rpm and 20 min, respectively. The fine powder was weighed for the processing of nanoscale materials and processed in the mixer mill with a frequency of 25/s and oscillation of 20 s for three repetitions. PBS was used to dissolve and prepare a 25 mg/mL stock solution, followed by 30 min of ultrasonication, autoclaving and finally storage at −20°C.

### Quality Control of the Active Components of *C. tubulosa* by HPLC

The ECH and VER contained gradient elution with octadecylsilane-bonded silica as filler, methanol as mobile phase A and 0.1% formic acid solution as mobile phase B. The detection wavelength was 330 nm. The ICA contained gradient elution with octadecylsilane-bonded silica as filler and acetonitrile water (30:70) as the mobile phase. The detection wavelength was 270 nm. The sample, control and negative control were measured as 10 μL each for the test.

### Cell Culture and MTT Assay to Measure Viability of MPP^+^-Treated Cells

MES23.5 cells were inoculated with 5% fetal calf serum, 1% glutamine, 2% 50× Sato’s solution and DMEM/F12 medium with 2% penicillin/streptomycin. They were incubated at 37°C in a 5% CO_2_ incubator with saturated humidity. The cells were isolated and passaged with 0.25% trypsin and the cell suspension was harvested in the logarithmic growth phase. Isolated cells with a density of 1 × 10^5^ were seeded into polylysine-coated 96-well plates, followed by the addition of different final concentrations (6.25, 12.5, 25, 50, 100, 200, 400 and 800 μmol/L) of MPP^+^ media. MES23.5 cells that were incubated with normal culture medium for 24 h and 48 h were used as negative controls in the *in vitro* experiments. The cells from the different treatment groups were incubated with MTT reagent for 4 h. The solution in the wells was subsequently discarded and 150 μL of DMSO was added and oscillated for 10 min. The absorbance of each sample at a wavelength of 570 nm was measured using an automatic microplate reader. The percentage of cell viability (%) = mean absorbance of the experimental group/mean absorbance of the negative control group × 100%.

Relevant concentrations of MPP^+^ medium were added for the 24 h treatment in MES23.5 cells using the same approach as for the *in vitro* culture. After the treatment, the solution in the well was discarded. Media containing different concentrations (10, 50, 100, 200, 250, 500 and 1000 μg/mL) of *C. tubulosa* nanopowder was added to the MES23.5 cells in different wells and left to incubate for 24 h and 48 h. The MES23.5 cells that were incubated with normal culture medium for 24 h and 48 h were used as negative controls. The MES23.5 cells that were incubated with MPP^+^ medium were used as the vehicle. The measurements were done in triplicate for each sample. The absorbance of the corresponding treatment and control groups was measured to calculate cell viabilities.

### TH Expression Measured by Immunocytochemistry

When *C. tubulosa* nanopowder was in 100, 200 and 250 μg/ml, the cell survival rate increased significantly (Figure 2G). Thus, in subsequent experiments, we tested the three concentrations: low-dose, middle-dose and high-dose groups. Three replications were tested for each of the *C. tubulosa* groups. Sterilized, polylysine-coated coverslips were placed into 6-well plates. Next, 5 × 10^4^ cells were seeded in each well and incubated for 24 h. Conventional fresh medium was replaced in the normal control group and a final concentration of 100 μmol/L MPP^+^ medium was replaced in the remaining treatment groups to incubate for 24 h. Conventional fresh media were then replaced in the normal control and vehicle groups, and final concentrations of 100, 200 and 250 μg/mL *C. tubulosa* nanopowder were incubated with the cells for 24 h in the low-, moderate- and high-dose *C. tubulosa* treatment groups, respectively. The MES23.5 cells in the different groups were washed three times in PBS to remove the supernatant and further fixed in 4% paraformaldehyde for 15 min. After washing with PBS, the cells were incubated with a peroxidase blocker at 37°C for 30 min and then washed again with PBS. A 0.2% Triton X-100 solution was used for cell permeabilization for 10 min, followed by a washing with PBS. Normal goat serum was added to each sample and incubated at room temperature for 30 min. The normal goat serum was then removed and primary antibody diluted 1:400 in PBS was added to each sample and incubated at 4°C overnight. The cells in the negative control group were incubated with PBS at 4°C overnight. After washing with PBS, the cells were incubated with biotin-labeled secondary antibody in the moisture chamber at 37°C for 20 min. Each sample was then washed in PBS and labeled with horseradish peroxidase-streptavidin conjugates (working solution C) at 37°C for 20 min. After washing with PBS, the cells were stained with DAB reagent in the dark for approximately 1–10 min and the development of a brown color was monitored under light microscopy. Each sample was then washed twice in distilled water for 1–2 min and the nuclei were counterstained with hematoxylin solution for 0.5–1 min. After thoroughly rinsing each sample in water, the cells were immersed in 1% hydrochloric acid alcohol for differentiation and 1% aqueous ammonia, followed by thoroughly washing the cells in water. The cells from each sample were then dehydrated in 70% ethanol for 2 min, 80% ethanol for 2 min, 90% ethanol for 2 min twice, 95% ethanol for 2 min twice and 100% ethanol for 2 min twice. The cells were then immersed in xylene solution for 2 min twice and mounted on a glass slide with neutral resins. Under light microscopy, each sample underwent image capturing and random selection of 5–10 effective visual fields to determine the expression of selected proteins in dopaminergic neurons indicated by the intensity of the brown particles and to semi-quantify the protein content by its average gray value.

### Apoptosis Rate of MES23.5 cElls Measured by Flow Cytometry

Adherent cells were washed once with PBS. For cell isolation, an appropriate amount of EDTA-free trypsin solution was added at room temperature and the solution was gently pipetted to allow adherent cells to detach. Cell culture medium was then added to stop the trypsinization. The mixture was transferred to a new centrifuge tube and then centrifuged 5 min at 1500 rpm to collect the isolated cells. After discarding the supernatant, the cell pellet was gently resuspended using PBS and the cells were counted. Approximately 1 × 10^5^ to 5 × 10^5^ resuspended cells were centrifuged for 5 min at 1500 rpm and the supernatant was discarded. Five microliters of Annexin V-FITC binding solution was added to the cell pellet to gently resuspend the cells. Another 5 μL of Annexin V-FITC was added and mixed thoroughly. Five microliters of propidium iodide solution were used for cell staining by incubating at room temperature for 10 min shortly before the flow cytometry.

### Western Blot Analysis for *in vitro*

The cells were divided into normal group, MPP^+^ treatment group, low-dose *C. tubulosa* treatment group, moderate-dose *C. tubulosa* treatment group and high-dose *C. tubulosa* treatment group. The expression of Bcl2 and Bax was measured in each. A total of 1 × 10^5^ cells per well in the 6-well plate were used for modeling and treatment in each group before cell harvesting. A mixed lysate, containing RIPA buffer, protease inhibitor and phosphatase inhibitor, was added to lyse the cells for 30 min on ice. After centrifugation, the supernatant was used for protein analysis. The total protein was quantified using a BCA assay and separated by 10% SDS-PAGE. The separated proteins were transferred to a membrane and incubated with a 5% skim milk blocking buffer at room temperature for 2 h. The membrane was then incubated in primary antibody (Bcl-2 0.34 mg/ml, Bax 0.11 mg/ml, 1:200 dilution) at 4°C overnight. After a washing step, the membrane was incubated in a secondary antibody (0.5 mg/ml, 1:5000 dilution) at 4°C for 1 h and then in ECL developer for 2 min for conventional development. Quantity One software was used for the semi-quantitative analysis of protein expression.

### Experimental Animal Modeling and Drug Administration

Fifty specific-pathogen-free 8-week-old male mice were randomly divided into five groups: a normal group, MPTP treatment group (Vehicle), low-dose *C. tubulosa* treatment group, moderate-dose *C. tubulosa* treatment group and high-dose *C. tubulosa* treatment group. The animals were housed at 20–22°C with free access to food and water. The mice in the normal group were intraperitoneally injected with an equal volume of normal saline for seven consecutive days. The mice in the other treatment groups were intraperitoneally injected with MPTP (30 mg/kg/d) for seven consecutive days to establish vehicles.

During PD modeling, the mice in the low-dose, moderate-dose and high-dose *C. tubulosa* treatment groups were intragastrically administered equivalent clinical volumes of 4 g/kg/d, 8 g/kg/d and 16 g/kg/d *C. tubulosa* nanopowder, respectively, for 14 consecutive days. The mice in the control and vehicle groups were intragastrically administered equivalent volumes of normal saline for 14 consecutive days. All experimental procedures were approved by the Ethical Committee of Fujian University of TCM and were performed according to the internationally accepted principles for laboratory animal use and care. All efforts were made to minimize animal suffering in this study.

### Behavioral Tests

#### Swimming Test (Zhu et al., 2014)

The coordination of body movement in mice was measured by Swimming test. The mice were individually placed in a water tank (25 cm in height and 10 cm in diameter) containing 10 cm of water and tested in a quiet environment to record their stationary duration over 5 min.

#### Open Field Test (Kawai et al., 1998)

Locomotor activity was measured by using the Open field test. The mice were tested in a quiet and dim lit environment and individually placed in a 30 cm × 30 cm × 15 cm transparent acrylic container with a 6 cm × 6 cm separation grid at the bottom. The mice were given 10 min to adapt to the environment and then the ambulation of the grid number and rearing frequency of individual mice were measured five consecutive times to obtain mean values.

### Brain Tissue Sampling

Before tissue sampling, the mice were fed *adlibitum* with free access to water and received drug intervention for 14 consecutive days. Four mice in each group were selected and quickly decapitated. The SN (Bregma: −2.75 mm −2.92 mm) from each animal was isolated and placed on ice. Brain tissues were rinsed with 0.9% ice-cold sodium chloride solution to remove any blood and dried on filter paper before storing at −80°C. Four mice from each group were anesthetized intraperitoneally and their chests opened. An infusion needle was then inserted into the left ventricle of each animal. To remove blood in the circulatory system, the right atrial appendage was cut and the animal was infused with 4°C normal saline until the liver turned pale to ensure successive perfusion. Once the effluent of the right atrium became clear, each animal was perfused with 4% paraformaldehyde fixative. After the perfusion, the brain tissue of each animal was then dissected carefully and post-fixed in 4% paraformaldehyde for 24 h. Fixed brain tissues were then rinsed under running water, dehydrated in a graded series of ethanol solutions and cleared in xylene solution. This was followed by paraffin immersion and embedding.

### Changes of DA Quantity Measured by HPLC

The nanopowdered SN from each group were placed in an ice bath containing 0.9% sodium chloride solution (1:9 ratio). The brain tissue was homogenized using an ultrasonic cell disruptor and centrifuged at 1200 rpm for 20 min at 4°C to obtain the supernatant. For HPLC, a Hypersil AA-ODS column (2.1 mm × 200 mm, 5 μm) at 30°C column temperature was used. Fluorescence detection was performed at 280 nm *λ*ex and 340 nm *λ*em. The injection volume was 10 μL.

### Expression of TH, GDNF, GFRα1 and Ret Detected by Immunohistochemistry

Paraffin sections (5 μm thick) of individual brain tissue were isolated from each animal and placed in a 40°C warm water bath for flattening and adhering to glass slides. All of the tissue slides were incubated in a 60°C oven for 3–6 h, followed by xylene dewaxing, gradient ethanol dehydration and antigen retrieval by incubating in a citric acid buffer and heating in a microwave for 20 min. The tissue slides were then incubated in a 3% H_2_O_2_ solution at room temperature for 10 min. After being washed three times in PBS, the tissue slides were incubated with normal serum in a closed chamber at room temperature for 20 min. Immunohistochemical staining was conducted according to the manufacturer’s instructions. A Motic Med 6.0 image analyzer was used to calculate the value of integrated optical density in the positively stained cells.

### Western Blot Analysis in Brain Tissues of Mice

This study assessed the protein expression of tyrosine hydroxylase (TH), GDNF, GFRα1, Ret, Bcl2 and Bax. The brain lysate from each group was homogenized for 30 min on ice, followed by low temperature centrifugation, 20,000 rpm, at 4°C for 5 min to collect the supernatant. The protein samples were separated under constant pressure using a 10% SDS-PAGE gel as described above. The primary antibody concentration: TH 0.15 mg/ml, GDNF 0.5 mg/ml, GFRα1 0.8 mg/ml, Ret 0.63 mg/ml, Bcl-2 0.34 mg/ml and Bax 0.11 mg/ml. The procedure was same as above.

### Statistical Analysis

This study used SPSS 20.0 statistical software for data processing and analysis. Parameter values were expressed as mean ± standard deviation ($\bar{x}$ ± S). ANOVA was used for single factor data analysis. LSD or Games-Howell test was used to compare the groups. *P* < 0.05 (or *P* < 0.01) was considered as a statistically significant difference.

## Results

### The Active Components of *C. tubulosa* Nanopowder

**Figure 1:**
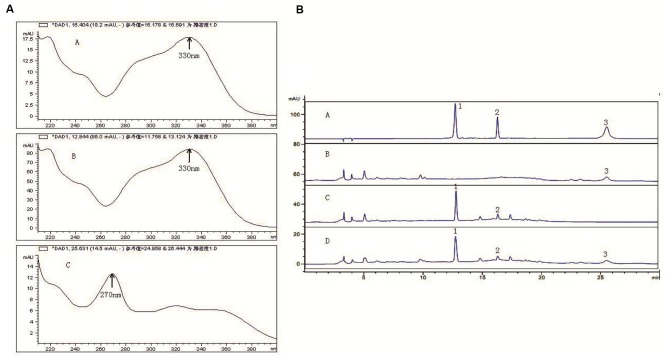
**Active components of *C. tubulosa* nanopowder.** Scanning wavelength of maximum absorption peak (**(A-A)** echinacoside (ECH); **(A-B)** verbascoside (VER); **(A-C)** icariin (ICA)) **(A)**; **(B-A)** mixed standard sample; **(B-B)** *C. tubulosa* negative sample; **(B-C)** Epimedium negative sample; **(B-D)** *C. tubulosa* nanopowder; 1: ECH; 2: VER; 3: ICA **(B)**.

In the range of 200–400 nm scanning, ECH in *C. tubulosa* and VER in 330 nm had the maximum absorption peak, which appeared within 20 min. ICA had maximum absorption peaks at 270 nm and appeared after 20 min (Figure 1A). The results showed that the negative samples did not interfere with the detection (Figure 1B). The samples and control had the same chromatographic peaks and the negative sample had none. This showed that the other ingredients in the sample did not interfere with the component being measured. Moreover, the three components and the adjacent peaks can reach the separation baseline and the separation degree was greater than 1.5.

### *C. tubulosa* Nanopowder Reduced MPP^+^-Induced Cytotoxicity in MES23.5 Cells

**Figure 2:**
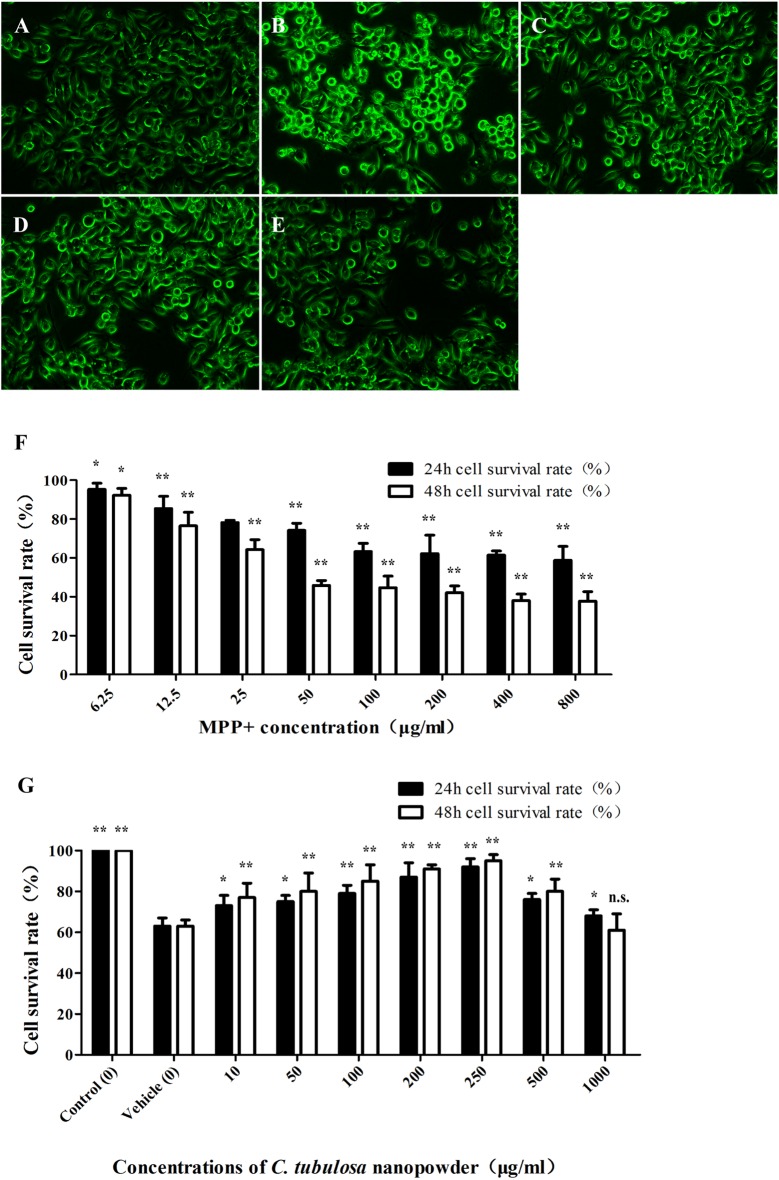
**Effect of *C. tubulosa* nanopowder on cytomorphology of MPP^+^-damaged MES23.5 cells.** The morphology of MES23.5 cells (**A**: control; **B**: vehicle; **C**: low-dose; **D**: middle-dose; **E**: high-dose). Effect of different concentrations of MPP^+^ treatment on viability of the MES23.5 cell line **(F)**. Effect of different concentrations of *C. tubulosa* nanopowder on viability of MPP+-treated MES23.5 cells **(G)**. Compare with the Vehicle: **P < 0.01; *P < 0.05; “n.s.” means no significance. *n* = 3.

The viability of MES23.5 cells was significantly reduced with increasing concentrations of MPP^+^. Figure 2F shows the significant cytotoxicity of different concentrations of MPP^+^.

*C. tubulosa* nanopowder reduced MPP^+^-induced cytotoxicity and enhanced the viability of MES23.5 cells. Figure 2G shows that *C. tubulosa* nanopowder dosages of 10–250 μg/mL exerted dose-dependent protective effects on the MPP^+^-treated MES23.5 cells.

### Cytomorphological Effect of *C. tubulosa* Nanopowder

Normal MES23.5 cells had good cell adhesion and were spindle shaped with clear cell boundaries and synapses. MPP^+^-damaged MES23.5 cells displayed poor cell adhesion and shrinkage, and many were suspended in the media with contracted synapses. These cells were aggregated, shrunken and round with vacuoles inside, and the nuclei were disintegrated or collapsed. *C. tubulosa* nanopowder in different dosages improved the cytomorphology of MES23.5 cells in different degrees by improving cell adhesion and synaptic clearance of the vehicle group. MES23.5 cells in the high-dose *C. tubulosa* treatment group showed morphology that was similar to the normal control group (Figures 2A–E).

### Effect of *C. tubulosa* Nanopowder on TH Expression and Apoptosis in the Cells

**Figure 3:**
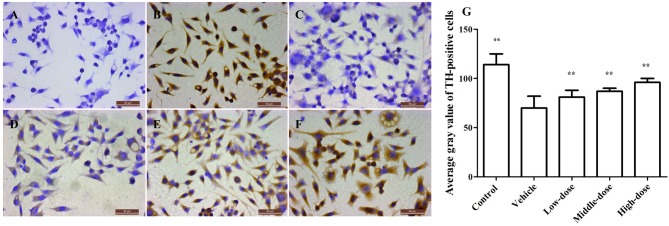
**Effect of *C. tubulosa* nanopowder on tyrosine hydroxylase (TH) in MPP^+^-damaged MES23.5 cells (400×).** The TH expression of MES23.5 cells by immunocytochemistry (**A**: negative control; **B**: control; **C**: vehicle; **D**: low-dose (100 μg/ml); **E**: middle-dose (200 μg/ml); **F**: high-dose (250 μg/ml)). Compare with the Vehicle: **P < 0.01. *n* = 3 **(G)**.

Figure 3 shows a significant reduction in the TH protein expression in the vehicle group. The TH protein expression increased differently in groups treated with different dosages of *C. tubulosa*. However, LSD test showed there was no significant difference between the three treated groups.

**Figure 4:**
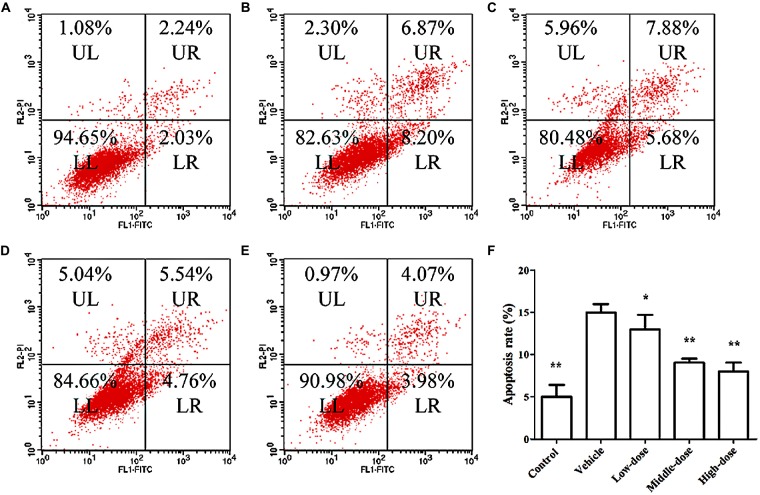
**Effect of *C. tubulosa* nanopowder on MPP^+^-induced apoptosis in MES23.5 cells. (A)** control; **(B)** vehicle; **(C)** low-dose (100 μg/ml); **(D)** middle-dose (200 μg/ml); **(E)** high-dose (250 μg/ml). UL: Upper Left (debris and damaged cells); UR: Upper Right (late apoptosis cells); LL: Lower Left (normal cells); LR: Lower Right (early apoptotic cells) **(A–E)**. Apoptosis rate = the number of (UR + LR) cells/total cell × 100%. Compare with the Vehicle: **P < 0.01; *P < 0.05. *n* = 3 **(F)**.

Figure 4 shows the results of the apoptosis assessment using flow cytometry. The rate of apoptosis in the vehicle group was significantly higher than in the other groups. Cells treated with different dosages of *C. tubulosa* nanopowder showed different degrees of decline in the apoptotic rate compared with the vehicle group. Cells in the middle- and high-dose *C. tubulosa* treatment group had the most significant improvement in apoptotic rate compared with the other *C. tubulosa* treatment groups. LSD test showed there was no significant difference between the two treated groups but a significant different between the low-dose group to them.

### Effect of *C. tubulosa* Nanopowder on Bcl2/Bax Protein Expression in the Cells

**Figure 5:**
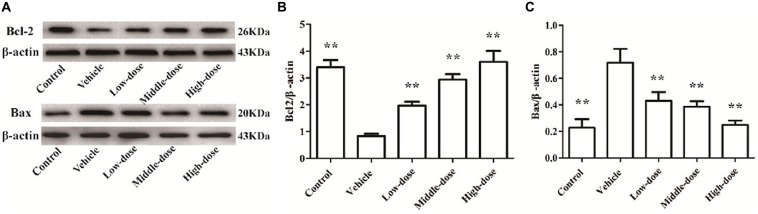
**Effect of *C. tubulosa* nanopowder on Bcl2/Bax protein expression in MPP^+^-treated vehicle MES23.5 cells.** The Bcl2 and Bax protein expression in MES23.5 cells **(A)**. The column charts of Bcl2 and Bax protein expression in MES23.5 cells **(B,C)**. Compare with the Vehicle: **P < 0.01. *n* = 3.

Figure 5 shows that the expression of Bcl2 protein in the cells of the vehicle group was significantly lower compared with the normal control group. In contrast, the expression of Bax protein in cells of the vehicle group was significantly higher than in the normal control group. *C. tubulosa* treatment groups showed increased Bcl2 protein expression and decreased Bax protein expression in MPP^+^-treated MES23.5 cells. Between three treated goups there were significant differences by LSD test. These effects were dose-dependent.

### Behavioral Tests

The results of the Swimming test suggested that the mice in the vehicle group had relatively long stationary durations, which increased over time. At day 14, the mice in the vehicle group had a significantly longer stationary duration than the mice in the normal control group. The stationary duration of the mice in the low-dose *C. tubulosa* treatment group was not significantly different from that of the mice in the vehicle group. However, the stationary duration of the mice in the high-dose *C. tubulosa* treatment group was significantly less than that of the mice in the vehicle group.

**Figure 6:**
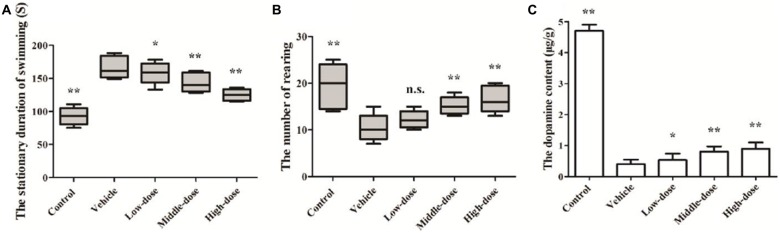
**Effect of *Cistanche tubulosa* nanopowder on the behavior and the dopamine content of the vehicle mice induced by N-methyl-4-phenyl-1,2,3,6-tetrahydropyridine (MPTP).** The stationary duration of swimming in Swimming test **(A)**. The number of rearing in Open field test **(B).** The dopamine content **(C)**. Compare with the Vehicle: **P < 0.01; *P < 0.05; “n.s.” means no significance. *n* = 3.

The results of the Open field test suggest that after MPTP-induced damage in the mice, the mice in the vehicle group demonstrated a significant decline in their ability for spontaneous activity as shown by the rearing frequency. After a 14-day administration of *C. tubulosa* nanopowder, the mice in the moderate- and high-dose treatment groups had significantly higher rearing frequencies compared with the mice in the vehicle group (Figures 6A,B).

### Effect of *C. tubulosa* Nanopowder on DA Content in Mice

Changes in the DA content of the SN were determined by HPLC. It was found that the DA content in the brain of the vehicle group was significantly reduced. The DA content in the brains of PD mice in the low-dose *C. tubulosa* treatment group did not differ significantly from the mice in the vehicle group. However, *C. tubulosa* treatment increased the DA levels in the brains of PD mice in a dose-dependent manner. The brains of PD mice treated with high-dose *C. tubulosa* had a significantly higher DA content than the brains of mice in the vehicle group (Figure 6C).

### Effect of *C. tubulosa* Nanopowder on TH Expression in Mice

**Figure 7:**
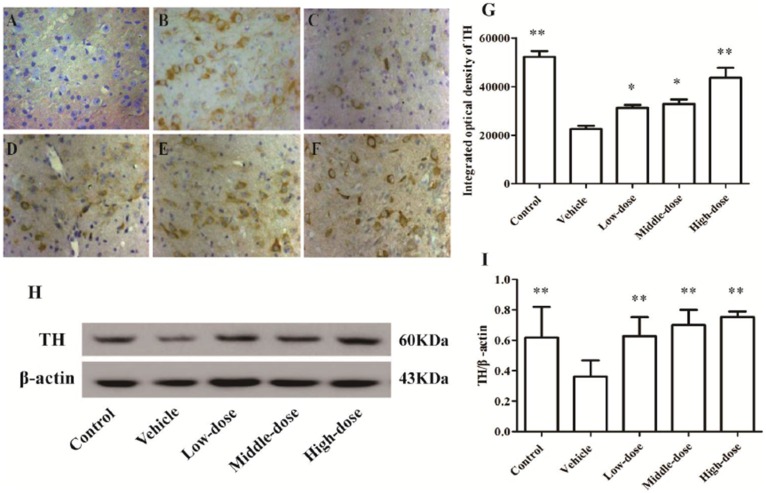
**Expression and integrated optical density of TH-positive cells in the substantia nigra (SN) in different treatment groups of mice (400×).** The TH-positive cells in the SN in different treatment groups of mice (400×) **(A–F)**. **(A)** Negative control; **(B)** Control; **(C)** Vehicle; **(D)** Low-dose; **(E)** Middle-dose; **(F)** High-dose. Integrated optical density of TH-positive cells in different treatment groups of mice **(G)**. Protein expression of TH **(H,I)**. Compare with the Vehicle: **P < 0.01; *P < 0.05. *n* = 3.

The number of TH-positive cells and the level of TH protein expression in the SN of MPTP-induced PD mice were lower compared with mice in the control group. After the *C. tubulosa* treatment, the number of TH-positive cells and the level of TH protein expression in the SN of MPTP-induced PD mice increased, with a significant difference between the high-dose *C. tubulosa* treatment group and the vehicle group by LSD test; and there were significant differences between the three treated groups (Figure 7).

### Effect of *C. tubulosa* Nanopowder on Protein Expression of GDNF and its Receptors, GFRα1 and Ret in Mice

**Figure 8:**
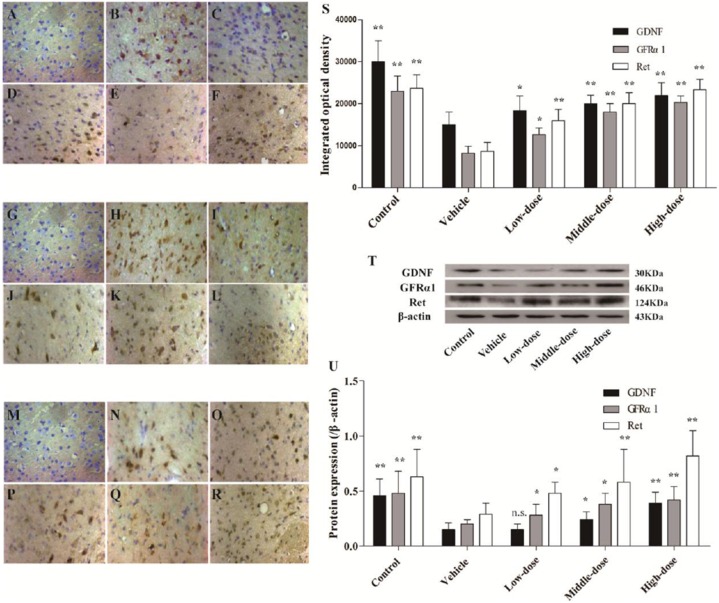
**Expression of glial cell-derived neurotrophic factor (GDNF) and its receptors, GDNF family receptor alpha (GFRα1) and Ret in different groups of mice.** The GDNF-positive cells in the SN in different treatment groups of mice (400×) **(A–F)**. The GFRα1-positive cells in the SN (400×) **(G–L)**. The Ret-positive cells in the SN (400×) **(M–R)**. **(A,G,M)** Negative control; **(B,H,N)** Control; **(C,I,O)** Vehicle; **(D,J,P)** Low-dose; **(E,K,Q)** Middle-dose; **(F,L,R)** High-dose. Integrated optical density of GDNF-positive cells, GFRα1- and Ret-positive cells, in different treatment groups of mice **(S)**. Protein expression of GDNF, GFRα1 and Ret **(T,U)**. Compare with the Vehicle: **P < 0.01; *P < 0.05; “n.s.” means no significance. *n* = 3.

The protein expression of GDNF and its receptors, GFRα1 and Ret, in the positively stained cells, was evaluated using immunohistochemistry. Western blot analysis was used to evaluate the protein expression levels in the SN of the different groups of mice. The findings for the different groups were similar using the two detection methods. The expression of GDNF and its receptor proteins, GFRα1 and Ret, in positively stained cells in the SN of the mice in the vehicle group was significantly lower than in the mice in the normal control group. Different dosages of *C. tubulosa* treatment increased the number of GDNF-, GFRα1- and Ret-positive cells (Figures 8A–S).

The protein expression of GDNF, GFRα1 and Ret in the SN of the mice in the vehicle group was significantly lower than in the mice in the control group. Increasing treatment concentrations of *C. tubulosa* nanopower significantly enhanced the expression of these proteins. The protein expression of GDNF, GFRα1 and Ret in the SN of the mice in the high-dose *C. tubulosa* treatment group was significantly higher than in the mice in the vehicle group (*P* < 0.01; Figures 8T,U).

### Effect of *C. tubulosa* Nanopowder on Protein Expression of Bcl2/Bax in Mice

**Figure 9:**
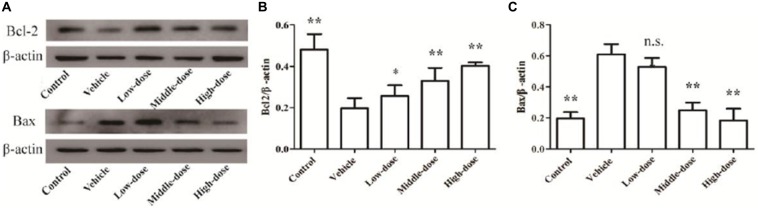
**Protein expression of Bcl2 and Bax in brain tissues from different treatment groups of mice.** The Bcl2 and Bax protein expression in brain tissues **(A)**. The column charts of Bcl2 and Bax protein expression in brain tissues **(B,C)**. Compare with the Vehicle: **P < 0.01; *P < 0.05; “n.s.” means no significance. *n* = 3.

Bcl2 protein expression was significantly reduced and Bax protein expression was significantly enhanced in the SN of mice in the vehicle group (*P* < 0.01) compared with the mice in the normal control group. High-dose *C. tubulosa* treatment significantly increased Bcl2 protein expression and significantly reduced Bax protein expression in the brains of the vehicle mice (*P* < 0.01; Figure 9). LSD test showed there was no significant difference between the middle-dose and high-dose groups but a significant different between the low-dose group to them.

## Discussion

### PD and Apoptosis

PD is a neurodegenerative disorder. According to Zhang et al. (2005), the prevalence is 10.7% in the Chinese population aged over 55 and 1.67% in those aged over 65. The number of PD patients has increased annually with the acceleration of global aging, placing a heavy financial burden on the families of patients and society at large.

In the brain, dopaminergic neurons are mainly involved in the synthesis and secretion of DA. They are widely distributed in the central nervous system and located primarily in the SN (80%). TH is the key rate-limiting enzyme for DA synthesis. Thus, the inhibition of TH activity reduces DA synthesis (Huot and Parent, 2007). The main pathological and biochemical changes of PD are apoptosis of dopaminergic neurons in the SN, a significant reduction of nigrostriatal DA and the formation of Lewy bodies in dopaminergic neurons (Dexter and Jenner, 2013). The etiology of PD involves associated genetic factors, environmental factors and aging of the nervous system (Allam et al., 2005). The pathogenesis of PD remains unclear in modern medicine. Since the late 1960s, levodopa replacement therapy has been used successfully to treat PD and has been recognized as a major turning point in PD treatment. However, the long-term application of this therapy causes side effects and the therapy does not treat the underlying causes of PD (Del Sorbo and Albanese, 2008). Therefore, active research for new drugs or treatment methods targeting the protection of dopaminergic neurons is crucial for treating PD.

In earlier studies, the application of terminal deoxynucleotidyl transferase-mediated dUTP nick end labeling indicated that 0.6%–4.8% of dopaminergic neurons in the SN of PD patients showed apoptosis (Mochizuki et al., 1996). Electron microscopy showed apoptotic features, including chromatic condensation and apoptotic bodies in dopaminergic cells (Anglade et al., 1997). Tompkins et al. (1997) performed an ultrastructural analysis of brain tissue autopsies from patients with PD, AD and diffuse Lewy body disease (DLBD). They found apoptotic bodies in the dense layer of the SN in PD and DLBD patients, providing conclusive evidence of neuronal apoptosis in PD and related diseases. Therefore, the reduction or suppression of apoptosis in dopaminergic neurons is fundamental for PD treatment.

Previous studies showed that MPTP induced PD-like symptoms. MPTP crosses the blood-brain barrier and is metabolized by type B monoamine oxidases in astrocytes. It is subsequently converted to toxic MPP^+^, which accumulates in the mitochondria of dopaminergic neurons through the protein intake of the DA transporter. It thus generates excess oxygen free radicals that inhibit complex I activity of the mitochondrial respiratory chain and ATP synthesis. These events further promote free radical formation and oxidative stress reactions, and eventually lead to the degeneration and death of dopaminergic neurons. Hence, this study used MPP^+^ to establish an *in vitro* vehicle in MES23.5 dopaminergic neurons and MPTP to induce a vehicle mouse for mutual verification. According to the results of the MTT assay, MPP^+^ significantly reduced the viability of MES23.5 cells, suggesting that MPP^+^ was cytotoxic to dopaminergic neurons. The results also demonstrated that *C. tubulosa* effectively enhanced the expression of anti-apoptotic proteins and inhibited the increase of MPP^+^-induced apoptosis.

### PD and GDNF

GDNF is a neurotrophic factor, which was first isolated by Lin et al. (1993). Lin et al. (1993) also showed that GDNF had specific nutritional effects on dopaminergic neurons in the midbrain of rats. GDNF, neurturin (NTN), persephin (PSP) and artemin (ART) constitute the GDNF family. They are structurally similar and functionally related secretory proteins (Kotzbauer et al., 1996; Baloh et al., 1998; Milbrandt et al., 1998; Woodbury et al., 1998).

The GDNF receptor consists of two components. The first component, GFRα, is fixed to the outer membrane of glycosylphosphatidylinositol (GPI) and anchored to the surface of connexin. The second component is Ret protein. Research has showed that there are four different types of GFRα: GFRα1, GFRα2, GFRα3 and GFRα4. GFRα1 is a high affinity receptor of GDNF (Onochie et al., 2000; Chen et al., 2001; Lindahl et al., 2001). Ret protein is a functional receptor of GDNF. The homodimer molecule of GDNF directly binds to GFRα1 to form complexes and interacts with Ret, resulting in the dimerization and activation of Ret. Due to autophosphorylation of Ret, Ret activates several common TH signaling pathways. In the absence of Ret protein, GDNF causes protein phosphorylation of MAPK, PI-3 and PLC-γ, in addition to mRNA expression and functional activity of C-fos through its receptor protein, GFRα1 (He et al., 2008).

Studies have demonstrated that GDNF had the strongest protective effect on dopaminergic neurons (Rangasamy et al., 2010; Campos et al., 2012). In vehicles using MPTP and 6-hydroxydopamine (6-OHDA) to induce damage in dopaminergic neurons, GDNF protects dopaminergic neuron by reducing apoptosis and promoting axonal growth to induce stem cell differentiation (Lucas et al., 2012; Littrell et al., 2013). Lin et al. (1993) showed that GDNF specifically promoted viability, differentiation and axonal growth of dopaminergic neurons to promote the uptake of DA in neurons. The study also showed that GDNF not only prevented acute toxicity, but also alleviated long-term toxicity of MPP+ or 6-OHDA in dopaminergic neurons, furthermore preventing cell death in stressed or damaged cells (Yu et al., 2010). In addition, GDNF promoted neural stem cell proliferation and differentiation toward dopaminergic neurons in the midbrain (Lindsay, 1995) to rescue dopaminergic neurons from retrograde degeneration (Hong-Juan et al., 2011).

Studies have shown that GDNF expression in the SN was significantly reduced in animal vehicles (Yang et al., 2010). This suggests that it may be one of the mechanisms of pathogenesis in PD rats. The injection of 5–15 μg/d GDNF into the lateral ventricle or striatum of an MPTP-induced vehicle animal for three consecutive months promoted nigrostriatal repair of the dopaminergic system in the vehicle animal (Grondin et al., 2002). Studies of GDNF treatment for a PD in animal vehicle have shown that intracerebral injection of GDNF into different brain regions, such as the SN, caudate nucleus and lateral ventricle, improved movement disorders associated with PD animal models, including decreased motor activity, muscle rigidity and tremor (Grondin et al., 2002). However, GDNF cannot directly pass through the blood-brain barrier. Therefore, local cerebral injection of GDNF involves substantial risk and difficulties in clinical application. Applications to introduce exogenous GDNF via controlled-release microspheres, sustained-release capsules and viral genes are still being studied (Liang et al., 2010; Yang et al., 2010; Qiao et al., 2012). Given the limitations of various techniques to introduce exogenous GDNF into the brain, neuroprotective agents that promote the release of endogenous GDNF are significant for clinical application.

### PD and *C. tubulosa* Nanopowder

PD is more common in middle-aged adults and the elderly. The theory of TCM considers that PD is primarily located in the brain and is mainly due to liver and kidney deficiency, in addition to vital energy and blood insufficiency. According to this theory, PD treatment should thus focus on invigorating kidney and bone marrow. Yang et al. (2010) used randomized, double blind and placebo-controlled clinical trials and found that combination therapy using Madopar and kidney-tonifying recipes alleviated the motor dysfunctions of PD patients. The treatment result was better than a single therapy using Madopar. Treatment efficacy of TCM monotherapy or compound prescription in PD has been confirmed in PD animal models and clinical applications. TCM applications for tonifying kidney and promoting blood circulation reduced the dosage of monotherapy for PD using Madopar. Some studies have suggested that TCM improved symptoms of PD and protected dopaminergic neurons, which might have been closely related to the promotion of endogenous GDNF expression (Hong-Juan et al., 2011; Qiao et al., 2012).

The kidney-tonifying compound used in this study, *C. tubulosa* nanopowder, contained *Cistanche*, epimedium and *Rhizoma polygonati*. Modern research suggests that the chemical composition of *Cistanche* is ECH, which protects dopaminergic neurons in the SN in the MPTP-induced PD mice, and inhibits the reduction of DA and the DA transporter (Zhao et al., 2010). In addition, it prevents 6-OHDA-induced reduction in DA and protects striatal dopaminergic neurons (Chen et al., 2007). Epimedium inhibits the activation of caspase-3 and exerts neuroprotective roles (Liu et al., 2011). Epimedium flavonoids effectively promote neural stem cell proliferation and differentiation (Yao et al., 2010).

In this study, *C. tubulosa* nanopowder antagonized the increase of MPP^+^-induced apoptosis in a dose-dependent manner. It significantly improved TH expression in the *in vitro* vehicle and had significant anti-apoptotic effects in dopaminergic neurons. The MPTP-induced vehicle mice showed behavioral disorders and significantly reduced TH expression in the midbrain tissues and DA levels, which are typical pathological features of PD. Different dosages of *C. tubulosa* nanopowder shortened the stationary duration, enhanced autonomous activities, improved behavioral disorders, elevated DA levels in the brain and increased TH expression in vehicles. These results suggested that *C. tubulosa* nanopowder exerted protective effects in dopaminergic neurons, thereby improving behavioral disorders of vehicles. Different dosages of *C. tubulosa* nanopowder increased the expression of GDNF protein and its receptor proteins in the brain of vehicle mice. High-dose *C. tubulosa* treatment significantly upregulated Bcl2 expression and reduced Bax expression, which suggested that *C. tubulosa* nanopowder might promote GDNF expression and secretion in the MPTP-damaged mouse brain. In addition, it might exert neuroprotective effects in dopaminergic neurons and minimize neuronal apoptosis through the neurotrophic support roles of GDNF.

This study demonstrated that *C. tubulosa* nanopowder exerted protective effects in dopaminergic neurons in both *in vitro* and *in vivo* and increased TH expression to improve DA content. It also improved behavioral disorders in an MPTP-induced vehicle mice, regulated protein expression of GDNF and its receptor proteins in the SN, and had anti-apoptotic effects in the PD mice. The mechanism underlying clinical effects of *C. tubulosa* nanopowder in PD may involve increasing the content of endogenous GDNF in the brain and thereby reducing the damage to dopaminergic neurons.

## Author Contributions

QX, WF are the co-first authors of the work, S-FY, Y-BC, WQ, S-YC participated in the experimental work, JC designed and guided the work.

## Conflict of Interest Statement

The authors declare that the research was conducted in the absence of any commercial or financial relationships that could be construed as a potential conflict of interest.
